# A Deep Convolution Method for Hypertension Detection from Ballistocardiogram Signals with Heat-Map-Guided Data Augmentation

**DOI:** 10.3390/bioengineering12030221

**Published:** 2025-02-21

**Authors:** Renjie Cheng, Yi Huang, Wei Hu, Ken Chen, Yaoqin Xie

**Affiliations:** 1 Shenzhen HUAYI Medical Technologies Co., Ltd., Shenzhen 518055, China; renjie926@foxmail.com (R.C.); huangyi@huayimt.com (Y.H.); huwei@huayimt.com (W.H.); 2 Shenzhen lnstitute of Advanced Technology, Chinese Academy of Sciences, Shenzhen 518055, China

**Keywords:** ballistocardiography signal, hypertension detection, deep learning

## Abstract

Hypertension (HPT) is a chronic disease characterized by the consistent elevation of arterial blood pressure, which is considered to be a significant risk factor for conditions such as stroke, coronary artery disease, and heart failure. The detection and continuous monitoring of HPT can be a demanding process. As a non-contact measuring method, the ballistocardiography (BCG) signal characterizes the repetitive body motion resulting from the forceful ejection of blood into the major blood vessels during each heartbeat. Therefore, it can be applied for HPT detection. HPT detection with BCG signals remains a challenging task. In this study, we propose an end-to-end deep convolutional model BH-Net for HPT detection through BCG signals. We also propose a data augmentation scheme by selecting the J-peak neighborhoods from the BCG time sequences for hypertension detection. Rigorously evaluated via a public data-set, we report an average accuracy of 97.93% and an average F1-score of 97.62%, outperforming the comparative state-of-the-art methods. We also report that the performance of the traditional machine learning methods and the comparative deep learning models was improved with the proposed data augmentation scheme.

## 1. Introduction

Hypertension (HPT), commonly characterized by resting systolic blood pressure (SBP) of 140 mmHg or higher, or diastolic blood pressure (DBP) of 90 mmHg or higher, affects a significant portion of the global adult population [1]. Cardiovascular disease, constituting 32% of global deaths, is primarily attributed to heart attacks and strokes (85% of cardiovascular-related fatalities) [2,3]. Approximately 1.28 billion people worldwide suffer from HPT, a condition that is responsible for approximately half of all heart diseases and strokes [3,4]. Nearly half of people with HPT globally are currently unaware of their condition [4], and less than 20% receive management [5]. In HPT patients, normal blood pressure may be observed briefly, but abnormalities can persist over 24 h ambulatory blood pressure (BP) monitoring, highlighting the primary challenge of requiring prolonged BP recording to diagnose the severity of HPT [6].

Various methods have been utilized for HPT monitoring. The traditional devices to assist individuals in measuring BP at home or in daily life typically employ oscillometric methods and utilize an inflatable upper arm cuff [7,8,9]. However, these methods do not provide continuous measurements [7]. Furthermore, wearing the cuff every time for BP measurement can cause discomfort to users [10]. Direct BP measurement methods overcome the limitations of traditional measurement techniques by completing BP measurements in every cardiac cycle, thereby monitoring BP changes more accurately. Although recognized as the gold standard, direct BP measurement requires arterial catheterization, which greatly limits its clinical application [11]. Non-invasive methods such as electrocardiography (ECG), photoplethysmography (PPG) signals, and signal-derived heart rate variability (HRV) are favored by researchers [5], but they still require the attachment of sensors directly to the subjects, remaining inconvenient for users.

**Figure 1:**
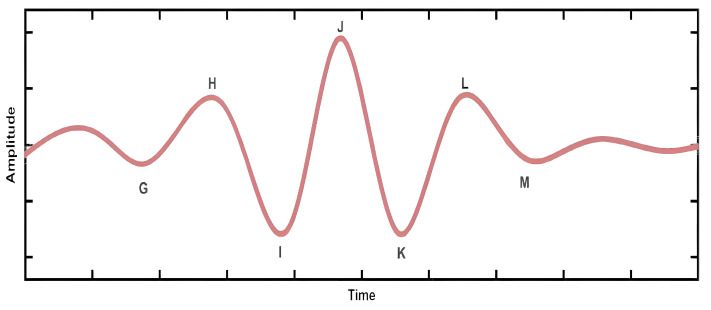
A waveform representing a heartbeat cycle in the BCG signal.

To overcome such drawbacks, a promising solution is to use ballistocardiography (BCG), a non-contact sensing technique. BCG generates a graphical representation of the repetitive motions of the human body, originating from the sudden ejection of blood into the great vessels with each heartbeat [5]. BCG signals are primarily generated by the heartbeat, and it is demonstrated that it can be utilized to evaluate cardiac performance during the cardiac cycle [12,13]. The cardiac cycle refers to the operation of the human heart from the beginning of one heartbeat to the beginning of the next heartbeat. It consists of two periods: one during which the heart muscle relaxes and refills with blood, called diastole, following a period of robust contraction and pumping of blood, called systole [14]. Figure 1 illustrates a typical example of one cycle of the BCG signal, where letters represent different segments of the BCG waves. The waveform from the G wave to the K wave represents the contraction period of the heart, corresponding to the QRS complex in the electrocardiogram signal [15,16]. The diastolic period is represented from the K wave to the end of the M wave. Therefore, by utilizing the systolic (G–K) and diastolic (K–M) periods in the BCG signal, physiological indicators such as heart rate and heart rate variability can be obtained for patients, indicating that BCG signals can be used to diagnose HPT [17]. The recording of human BCG signals benefits from the non-contact positioning of sensors on mattresses or beds where individuals sleep or sit [16].

Several machine learning and deep learning methods have been proposed for HPT classification with BCG signals, but it still remains a challenging task. Rajput et al. [18] used manually extracted nonlinear features from BCG signals for automated HPT detection and employed an ensemble gentleboost (EGB) classifier, achieving an accuracy of 89.00%. Liu et al. [19] employed class association rules (CARs) to classify BCG signals for HPT patients, achieving the highest detection accuracy of 85.2%. The machine learning methods deeply rely on manual feature selection, resulting in insufficient accuracy and generality. Gupta et al. [20] proposed a fully automated HPT detection system using time–frequency spectral images and a CNN (convolutional neural network), achieving an accuracy of 97.65% in detecting HPT from BCG signals. Although the deep learning models show the powerful capability of automated and self-adaptive feature extraction, their inherent complexity coupled with the computational demands constrain their implementation in clinical practice. Moreover, as time-series signals, extracting multi-scale features from BCG signals can provide more discriminative information for HPT detection by capturing both global and local information at different scales. Additionally, the interpretability of deep learning models for HPT detection using BCG signals has not been sufficiently studied, and understanding the extracted features through interpretability techniques could help to clarify how the model distinguishes between HPT and normal subjects, as well as improve the performance of the models.

To address such limitations, in this paper, we propose a novel and lightweight end-to-end CNN-based deep learning network called BH-Net. This network leverages a residual network structure to extract features at different scales, which are then fused for final classification. Additionally, we utilize the feature map visualization technique to interpret the correlation between the features extracted by the network and the properties of BCG signals. This interpretation guides a data augmentation process to generate enhanced data-sets, thereby improving the classification accuracy.

Consequently, the contributions of this study can be outlined as follows:

(1) We introduce BH-Net, a novel BCG signal HPT classification system. In comparison to other deep learning models, BH-Net demonstrates superior classification performance with fewer learnable parameters, leading to faster and more efficient computation.

(2) We propose a data augmentation scheme to generate enhanced sample sets by selecting the J-peak neighborhood of the BCG time sequences to improve the classification accuracy based on the analysis of the heat-map generated by the proposed model.

The rest of the paper is organized as follows. Section 2 summarizes the related work. Section 3 introduces the materials and methods in detail. Section 4 displays the experiments and results. Section 5 presents the discussion. Section 6 concludes the paper.

## 2. Related Work

### 2.1. Hypertension Classification Based on Physiological Signals

Various physiological signals are employed to monitor HPT. When compared to HPT patients, the amplitude of the HRV signal spectrum is greater in a healthy population and can serve as a diagnostic tool for HPT symptoms [21,22]. HRV signals represent continuous heartbeats, which can usually be extracted from the PPG signal [23] and ECG signal [24]. After denoising [25], ECG signal and PPG signal waveforms can also be directly employed for HPT detection. The ECG records the electrical potentials on the body surface originating from the heart, and these signals can offer information on the rhythm, structure, and function of the heart [26,27]. Through advanced analysis, the ECG signals in individuals with HPT can be correlated with BP measurements and may even discern higher clinical risk [28,29]. PPG detects blood volume changes using photoelectric techniques (transmissive or reflective) to form a signal that encompasses veins, arteries, and capillaries [30]. The PPG signal can extract features related to blood pressure and can be used to detect HPT [31,32].

Researchers have recently introduced various artificial-intelligence-based methods, particularly machine learning and deep learning techniques, for continuous monitoring and classification of HPT using ECG, PPG, and HRV signals. Parmar et al. [33] processed ECG signals using Fourier decomposition and cosine-modulated filters. Subsequently, they classified HPT patients through integrated classifiers such as K-Nearest Neighbor (KNN), achieving a maximum accuracy of 99.99%. Gupta et al. [34] employed flexible analytic wavelet transform (WT) for the screening of HPT using ECG signals. They successfully detected HPT patients with an accuracy of 96.60% utilizing an ensemble bagged tree classifier. Simjanoska et al. [35] introduced an ECG-based HPT classification system that utilizes wearable physiological sensors for acquiring ECG signals, demonstrating an impressive classification accuracy of 96.68%. Martinez-Ríos et al. [36] proposed the wavelet scattering transform method to extract features from PPG data combined with clinical data for early HPT detection, achieving 71.42% accuracy with a Support Vector Machine (SVM) classifier. Tanc et al. [37] presented an automatic HPT classification and early diagnosis technique using PPG signals based on Synchrosqueezing Transform and a pretrained convolutional neural network (GoogLe-Net), achieving a 96.83% F1-score. Evdochim et al. [38] developed an HPT diagnosis tool that combines PPG signal morphology analysis with an SVM Gaussian model to improve blood pressure class detection, achieving up to 72.9% accuracy. Alkhodari et al. [39] utilized demographic characteristics and manually extracted HRV signal features to predict high-risk BP patients. This was accomplished through a model based on decision trees and random undersampling boosting (RUSBOOST), resulting in an accuracy of 81.25%. Kirtana et al. [40] proposed a remote HRV variability monitoring system for HPT patients, which acquires HRV signals via a pulse sensor and transmits them wirelessly, thereby enabling remote health monitoring. Ni et al. [41] employed an SVM for detecting HPT using HRV signals obtained from an overnight waist belt continuous sensing system, achieving an accuracy of 93.33%.

However, the accurate acquisition of ECG, PPG, and HRV signals currently heavily relies on contact-based sensors, which may limit application [42,43]. Therefore, this study utilizes BCG technology, which enables non-contact acquisition of signals.

### 2.2. Hypertension Classification Based on BCG

A BCG signal can be acquired in a non-contact manner and has been demonstrated as an effective screening tool for HPT [44]. Concurrently, sensors designed for measuring BCG signals are strategically positioned regarding areas such as mattresses, pillows, chairs, beds, or even weighing scales, enhancing the convenience and comfort of BCG signal monitoring for HPT [16]. HPT can lead to increased cardiac load, resulting in abnormal changes in cardiac electrophysiology and autonomic nervous activity, which are detectable in the BCG signal [16,44]. Kitt et al. [45] advocated for the potential of home monitoring through BCG signals in enhancing the detection and management of HPT. Lin et al. [46] examined recent advancements in various physiological signal monitoring, particularly focusing on sensors based on diverse mechanisms and principles, with an emphasis on BCG signal monitoring.

Nowadays, researchers have proposed various methods based on artificial intelligence (AI), especially machine learning and deep learning approaches, to continuously monitor, manage, and classify HPT through BCG signals. For monitoring HPT patients using BCG signals, Rajput et al. [18] introduced an automated HPT detection system. They applied empirical mode decomposition (EMD) and wavelet transform (WT) to extract nonlinear features from the BCG signals, achieving an accuracy of 89.00%. The ensemble empirical mode decomposition (EEMD) method was applied by Song et al. [47] to BCG signals, and various cardiovascular disease-based features were extracted. Their method demonstrated the ability to detect HPT with an accuracy of 92.3%. Liu et al. [19] employed CARs to distinguish the HPT class from normal using the same data-set, achieving the highest detection accuracy of 85.2%. Seok et al. [48] introduced a BP monitoring system that utilizes a CNN and two-channel BCG signals. The findings substantiate that the proposed model accurately estimates the rapidly rising BP during the recovery state. Rajput et al. [6] employed continuous wavelet transform (CWT) scalograms and a 2D-CNN model for the classification of HPT subjects with an accuracy of 86.14%.

Machine learning techniques encounter challenges including labor-intensive manual feature extraction, lower model classification accuracy, and limited model generalization capability. Although deep learning techniques address these issues to some extent, they typically improve classification accuracy by increasing model complexity while raising the cost of the modeling process, posing challenges for embedded monitoring systems. To address the aforementioned challenges, in this study, we proposed a deep learning model with high accuracy, fast inference speed, and low memory usage. We also applied a visual explanation technique to analyze the interpretability of the proposed model.

## 3. Materials and Methods

**Figure 2:**
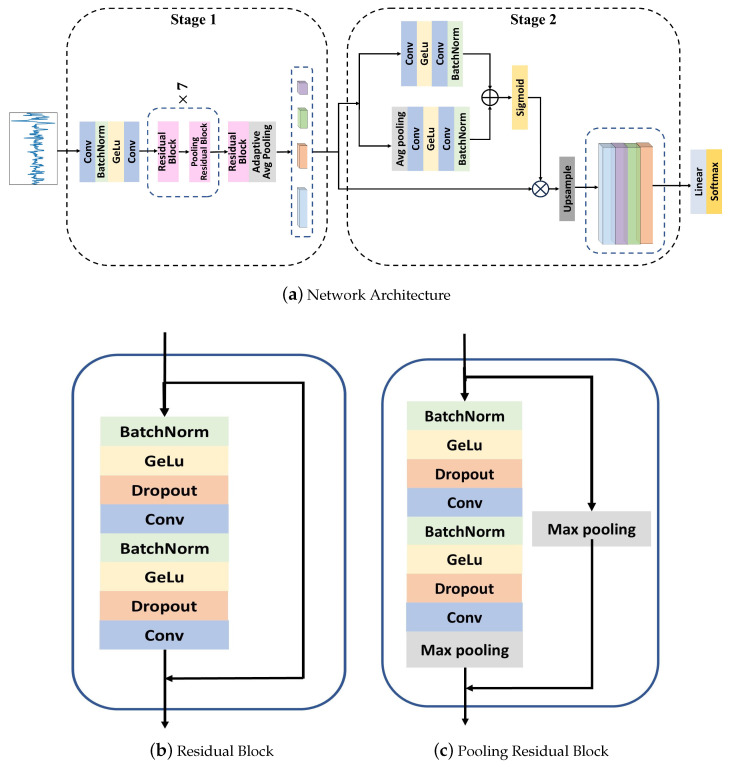
(**a**) The architecture of the proposed BH-Net; (**b**) the residual block structure in BH-Net; (**c**) the pooling residual block structure in BH-Net.

In this section, we will describe the proposed deep learning scheme BH-Net for HPT classification from BCG signal samples in detail, as shown in Figure 2. We introduce the Gradient-Weighted Class Activation Mapping (Grad-CAM) scheme to interpret the deep learning result. We also introduce a data augmentation scheme based on the model interpretability to improve classification accuracy.

### 3.1. BH-Net

To enhance the precision of HPT detection with BCG signals, we introduce an innovative end-to-end model, BH-Net, whose architecture is depicted in Figure 2a. The model comprises two key stages: the first stage, feature extraction, and the second stage, feature fusion. The feature extraction stage aims to generate features of diverse scales from the BCG signals. The feature fusion stage aims to integrate these multi-scale features to improve the efficiency and accuracy of the classification of HPT patients and healthy control groups.

#### 3.1.1. Feature Extraction

CNNs have been proven to be highly effective in physiological signal classification tasks, demonstrating the ability to learn hierarchical representations of signal data [49]. To address the issues of gradient vanishing and exploding, variant residual structures have been proposed. The shortcut connections between layers facilitate the well-propagation of information in the networks, thereby enhancing feature reuse and enabling the training of deeper networks. Inspired by the residual architecture proposed by Hannun et al. [50], we introduce two residual block structures, depicted in Figure 2b,c. The residual block comprises two repeated sequences of Batch Normalization, a GELU activation layer, a dropout layer with a probability of 0.1, and a convolutional layer with zero padding. The convolutional layer uses a kernel size of 17×1, a stride of 1, and the number of filters starts at 16. After every two residual blocks, the number of filters increases to 32, 48, and 64, respectively. Through skip connections, the input to the block is element-wise added to the output of the last convolutional layer, forming the output of the residual block. For the pooling residual block, a max-pooling layer is applied to the output of the residual block for down-sampling. The pooling residual block applies a max-pooling layer for down-sampling after each residual block. For pooling residual block, the convolutional layers use a kernel size of 17×1, a stride of 1, and the number of filters starts at 16 for the first block, then increases to 32, 48, and 64 for every two blocks, respectively.

The signals first pass through two convolutional layers, both with a kernel size of 19×1, 16 filters, a stride of 2, and a Batch Normalization layer followed by a GELU activation function interposed between them. The preliminary extracted features enter 7 stacks composed of a residual block and a pooling residual block. An extra residual block follows the last stack. Each pooling residual block includes a max-pooling layer with a kernel size of 2 and a stride of 2 to perform down-sampling operations, resulting in a final feature map sub-sampled by a factor of $2^{7}$ from the original input size. To enhance the ability of the network to capture features under multiple scales, we introduce Adaptive Average Pooling layers to obtain 4 feature maps of different scales.

#### 3.1.2. Feature Fusion

To integrate feature information of different scales and capture the features of macro- and micro-body motions, we introduce a Multi-Scale Channel Attention Module inspired by Dai Y et al. [51] for feature fusion.

The feature fusion module is composed of two branches, provided by (1)–(3).

The first branch extracts the global channel context ω by squeezing the feature map into a scalar, endowing the model with an understanding of global context features and large-scale motions. The feature map *X* obtained in the feature extraction phase initially undergoes a Global Average Pooling (GAP) process, calculating the average value of each channel in the feature map, followed by two convolutional layers of 32 and 64 filters, respectively, with a kernel size of 1×1, and a stride of 1.Then, the global feature context is calculated via a bottleneck structure as denoted by (1).

The second branch extracts the local channel context L of the input feature map *X*, as detailed in (2). Similarly, the second branch employs two convolutional layers with the same parameters as the first branch. The local channel context maintains the same dimensions as the input and pays more attention to the features of micro-movement.

Ultimately, the global channel context ω and local channel context L are combined via a broadcasting addition. These features then undergo a sigmoid activation function to generate attention weights. This attention weight is point-wisely multiplied with the input feature map *X*, resulting in the refined feature vector $X^{\prime }$, as shown in (3).

(1) $$\omega =B\left(\mathrm{Conv}\left(G\left(\mathrm{Conv}\left(g(X)\right)\right)\right)\right)$$

(2) $$L=B\left(\mathrm{Conv}\left(G\left(\mathrm{Conv}\left(X\right)\right)\right)\right)$$

(3) $$X^{\prime }=X⊗\sigma \left(\omega ⊕L\right)$$

where g(X) represents the GAP, Conv signifies convolution, *G* stands for the GELU activation function, *B* denotes Batch Normalization, and σ represents the sigmoid function.

After the feature fusion module, the refined feature vectors of the 4 different scale feature maps are up-sampled using bi-linear interpolation to match the length of the longest feature vector and then concatenated to form the final fused feature vector. The fused feature vectors are mapped to different categories through a classification head. The classification head comprises fully connected layers and a SoftMax activation function, responsible for mapping the learned representations from the feature extraction module to category probabilities.

### 3.2. Heat-Map-Guided Data Augmentation

Selvaraju et al. [52] introduced the Grad-CAM technique for image classification problems. Grad-CAM is capable of creating visual explanations for any CNN without requiring architectural changes or retraining, providing more insight into the behavior of the models by visualizing a heat-map representing where the model aims to make certain decisions during detection or prediction tasks.

**Figure 3:**
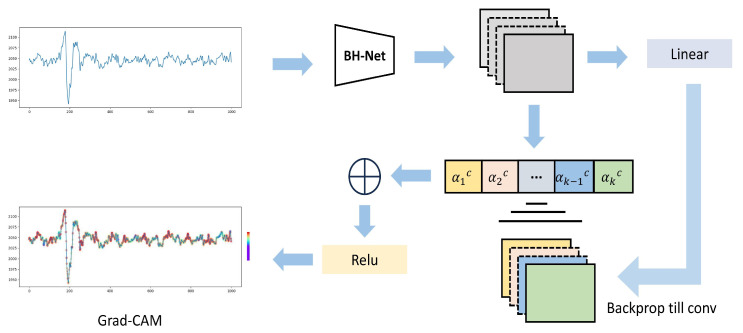
BH-Net with Grad-CAM architecture diagram (red indicates higher weights, purple indicates lower weights).

**Figure 4:**
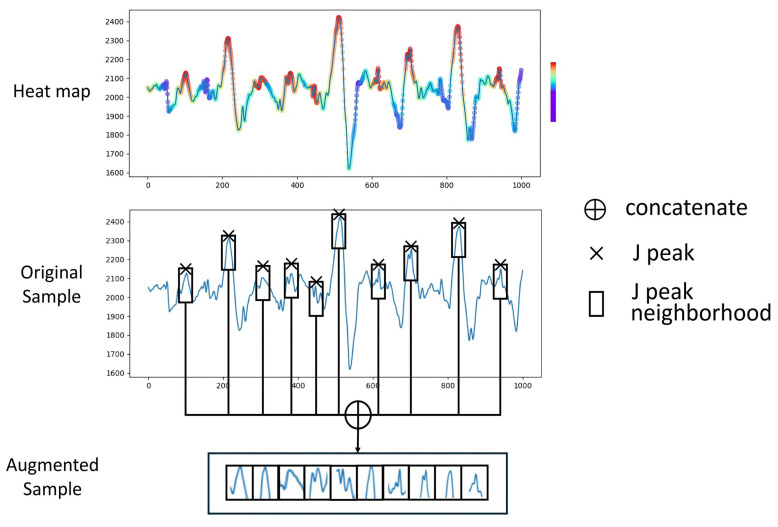
Heat-map-guided data augmentation scheme (red indicates higher weights, purple indicates lower weights).

In a similar manner, we adopted the Grad-CAM for the BCG time sequences to obtain a heat-map to visually demonstrate which part of the BCG signal contributes more for the HPT decision made by the proposed model. Figure 3 illustrates the complete process of applying the Grad-CAM algorithm to BH-Net. Grad-CAM can be applied to any convolution layer, but usually the last layer is chosen for implementation. By comparing the BCG signal sample and the corresponding heat-map, we qualitatively observe that there is a correlation between the high-weight regions and the J-peak neighborhoods. A typical heat-map and the original sample are shown in Figure 4.

Based on this observation, we proposed a data augmentation scheme for HPT detection from BCG signals. For an arbitrary unclassified BCG sample, the sample was z-score-normalized, and the J-peak points were located by comparison of neighboring values within a localized range. Regions of certain radius around the J-peak points are considered to be potentially significant and to contain more information for the HPT decision making process and therefore were picked and concatenated to generate the augmented sample to enhance the feature representation, as shown in Figure 4. Various deep models and classification methods will be tested with the original data-set and the augmented data-set to verify the effect of the proposed data augmenting scheme.

## 4. Experiments and Results

### 4.1. Implementation Details

In this study, we employed a public data-set [15] to rigorously evaluate the proposed method. This data-set includes the BCG signals from 128 subjects, originating from continuous recordings of BCG signals from the participants in the sleep state over a full-night duration of approximately 10 to 13 h, with a sampling frequency of 100 Hz. The data-set contains BCG signals from 67 healthy control subjects and 61 HPT patients. To reduce computational times, the BCG sequences from the data-set were divided into fixed-length samples of 10 s each. In total, we obtained 398,935 BCG signal samples, where 184,631 samples are from HPT subjects and 214,304 BCG samples are from healthy control subjects. Additionally, z-score normalization was applied to all the BCG signals. Table 1 presents detailed information about the BCG database. For neural network training and testing, we adopted a 9:1 training-to-testing split and used a 10-fold cross-validation scheme to evaluate the model, with the final results obtained by averaging the validation metrics across all the folds.

The model was configured with a batch size of 128 and incorporates Early Stopping technology, which ceases training if the validation performance fails to improve for ten consecutive iterations. The selected optimizer is Adam [53], and the loss function is cross-entropy, with a learning rate set to 0.00001. To mitigate over-fitting, the dropout rate in BH-Net is established at 0.1. The model is implemented with the PyTorch framework, on an Nvidia RTX 3090 24 GB GPU workstation.

### 4.2. Hypertension Classification Performance

We employed a set of comprehensive standard metrics to evaluate the classification results, including the Receiver Operating Characteristic Area Under the Curve (ROC-AUC), accuracy, average Macro-F1-score, sensitivity score, specificity score, and precision score. Positive cases (*P*) denote the count of HPT samples, whereas negative cases (*N*) represent the count of healthy control samples. True positive (TP) signifies the number of HPT samples accurately identified by the model, whereas true negative (TN) denotes the count of healthy control samples correctly classified by the model. False positive (FP) indicates the number of healthy control samples that are erroneously classified as HPT by the model, while false negative (FN) represents the count of samples that are HPT but are incorrectly classified as healthy controls by the model.

(4) $$\mathrm{Accuracy}=\frac{TP+TN}{P+N}$$

(5) $$\mathrm{Specificity}=\frac{TN}{TN+FP}$$

(6) $$\mathrm{Precision}=\frac{TP}{TP+FP}$$

(7) $$\mathrm{Sensitivity}\left(\mathrm{Recall}\right)=\frac{TP}{TP+FN}$$

(8) $$\mathrm{Macro-F1-Score}=\frac{2\times \mathrm{Precision}\times \mathrm{Recall}}{\mathrm{Precision}+\mathrm{Recall}}$$

We report that the proposed BH-Net achieves an ROC-AUC of 98.43%, accuracy of 97.93%, Macro-F1-score of 97.62%, sensitivity of 97.77%, specificity of 97.04%, and precision of 98.23%.

We also compare the proposed method with the following state-of-the-art comparative techniques:

(1) Traditional machine learning methods, including decision trees (DTs), K-Nearest Neighbor (K-NN), Support Vector Machines (SVMs), and Random Forest (RF), have been widely applied in the detection of HPT through physiological signals [36,54].

(2) General-purpose deep network structures include ResNet34 [55], ResNet50 [55], VGG16 [56], and AlexNet [57].

(3) Several state-of-the-art methods have been developed for the automated detection of HPT using BCG signals, including those by Gupta et al. [58], Rajput et al. [6], and HYP-Net [20]. Among these, HYP-Net, specifically designed for HPT detection, has demonstrated to be the most effective model when applied to this public data-set.

**Figure 5:**
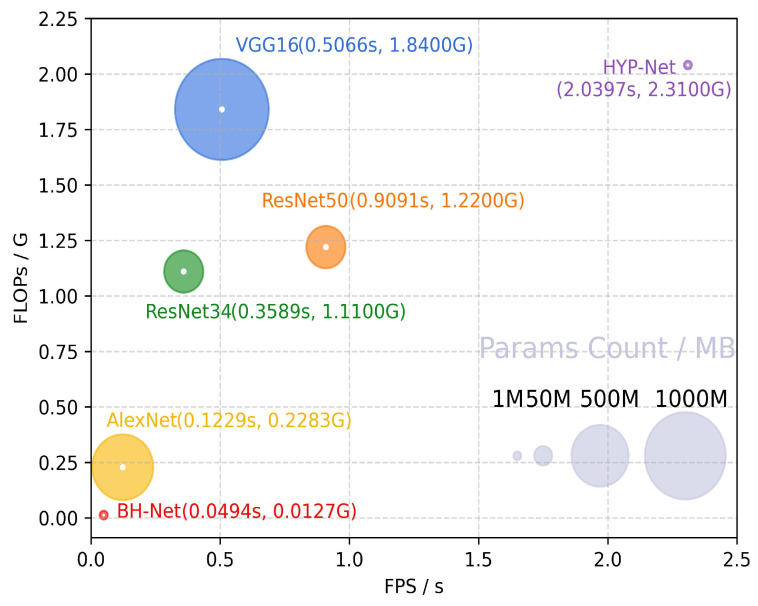
Comparison of the parameter counts, FLOPs, and inference times of different models.

We show a detailed comparison in Table 2. We can see that the deep network significantly outperforms the traditional machine learning methods. The traditional machine learning methods often require expert knowledge to design handcrafted features and rely on static features, limiting their ability to capture temporal dependencies and dynamic patterns in time-series signals. The proposed network outperforms the general-purpose deep networks. The traditional deep learning models focus on local features in BCG signals but lack integration of multi-scale local and global information. We report that the calculation efficiency of the proposed BH-Net demonstrates advantages over the HYP-Net. The proposed method only required 0.0494 s/frame inference time, while HYP-Net required 2.0397 s/frame. The inference time, FLOPs (floating-point operations per second), and parameter counts for all the comparative deep models were evaluated under the same hardware and software implementations and summarized in Figure 5 and Table 2. Table 3 compares our method with state-of-the-art approaches for automated HPT detection using BCG signals. It summarizes each method, their input formats, and accuracy, demonstrating that our proposed model outperforms the others.

### 4.3. Classification Results with Augmented Data

From Section 4.2, we can see that, due to the complexity of the BCG time sequences, the ability of the traditional classification methods to recognize the features of the HPT signal is not sufficient, resulting in relatively low accuracy.

**Figure 6:**
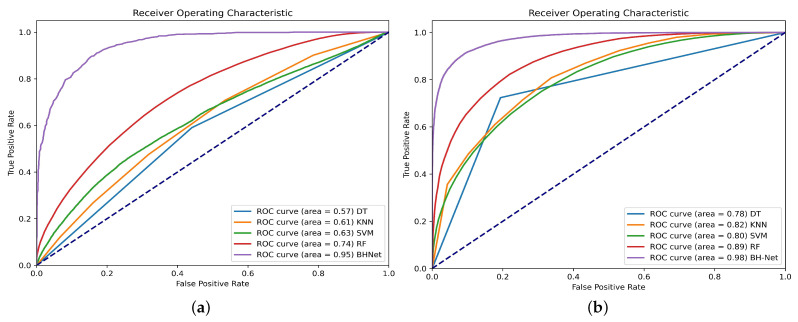
ROC curves for different machine learning models: (**a**) ROC curves with the original data-set; (**b**) ROC curves with the augmented data-set.

We used the augmented data-set described in Section 3.2 to retrain the above-mentioned methods. Compared with the original data-set, there is noticeable improvement in performance regarding all the methods. All the accuracy metrics for the machine learning models are improved by around 20%, as shown in Table 4. The RF model continues to lead in accuracy performance, surpassing the other models in accuracy, macro-average F1-score, sensitivity, specificity, and precision, achieving a level around 85%, with a particularly high ROC. The ROC curves with the original and augmented data-sets are summarized in Figure 6.

**Figure 7:**
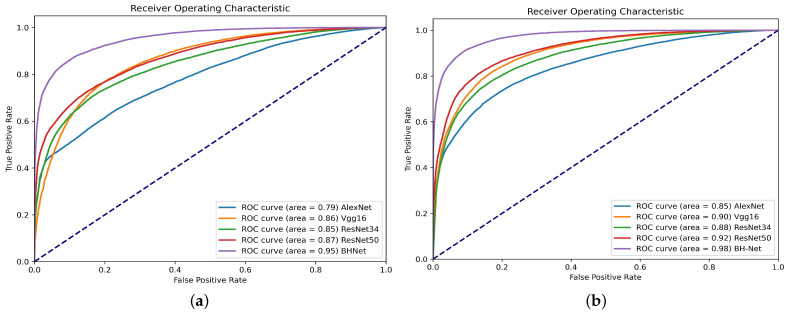
ROC curves for different deep learning models: (**a**) ROC curves with the original data-set; (**b**) ROC curves with the augmented data-set.

We also retrained the general-purpose deep learning models evaluated in Section 4.2. The HYP-Net network used frequency domain spectrogram images of the BCG signal as the input; therefore, the proposed data augmentation scheme is not applicable. We also observed significant accuracy improvement. The proposed BH-Net continues to lead in accuracy. We report that an accuracy rate of 97.93%, a macro-average F1-score of 97.62%, a sensitivity of 97.77%, a specificity of 97.04%, and a precision of 98.23% are achieved. For other deep models, a general improvement around 5% can be observed. The accuracy metrics for the deep learning models are summarized in Table 5, and the ROC curves are presented in Figure 7.

Therefore, we can conclude that the proposed data augmentation scheme is effective in improving the HPT detection accuracy. The J-peak neighborhood regions are demonstrated to contain more information that is highly correlated with the HPT BCG features than other parts of the time sequence. We can also infer that heat-maps of high-precision deep models can provide prior knowledge to enhance feature representations.

## 5. Discussion

### 5.1. Analysis of Hypertension Classification Results

In this study, we evaluated various learning methods for HPT detection with BCG signals. Traditional machine learning methods typically rely on prior knowledge to design and select handcrafted features, which requires expertise. Also, the traditional methods rely more on static features, thus lacking effective modeling capabilities to capture the temporal dependencies and the dynamic patterns in time-series signals. The SVM method handles nonlinear problems with kernel functions [59]. The KNN method is based on distance metrics [60], and the DT and RF methods handle multidimensional features by performing splits [61,62]. We can see that the above-mentioned traditional machine learning methods are not specifically designed for time-series data and do not take the temporal relationships within the data into account. Due to the distinctive and complex temporal characteristics of BCG signals, extracting these dynamic features through handcrafted methods is difficult, making it challenging for traditional methods to effectively capture key information in the signals, resulting in unsatisfactory classification accuracy. However, it is worth mentioning that traditional machine leaning methods usually show advantages in calculation efficiency, model complexity, and hardware requirements, and they may be preferred for clinical scenarios with high real-time calculation requirements, providing a suitable data augmentation scheme to enhance feature representation and improve the accuracy performance.

Deep learning models have been proven to be more effective than traditional machine learning methods, especially with their self-adaptive feature extraction ability. Several classical deep networks have been evaluated in this study. The results show that the deep models all outperform the machine learning methods significantly. The proposed BH-Net leads the accuracy performance by achieving an accuracy of 97.93%, a Macro-F1 of 97.62%, a sensitivity of 97.77%, a specificity of 97.04%, and a precision of 98.23%. BCG signals, as a type of physiological signal, are typically time-series data with strong temporal dependencies and periodicity. Feature extraction from BCG signals not only requires capturing short-term local fluctuations, such as the waveform characteristics of each heartbeat, but also understanding the global patterns between heartbeats. Traditional deep learning models may focus on capturing local features within BCG signals but are less effective at integrating both local and global information through multi-scale feature integration. On the other hand, the proposed network, BH-Net, pays specific attention to the extraction and integration of multi-scale features, making it more suitable for time series classification tasks.

**Figure 8:**
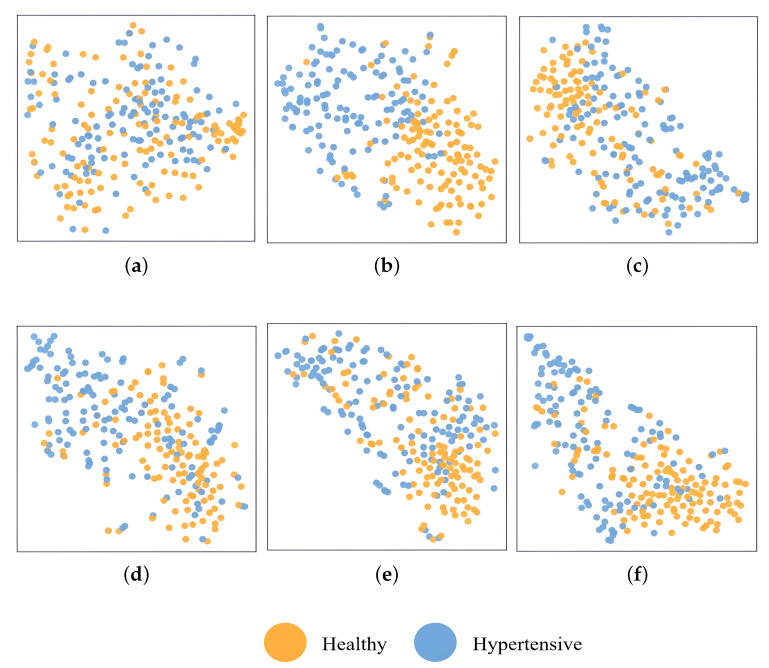
The visualization of input BCG signals and feature maps before the fully connected layers of each network through t-SNE (orange and blue denote the healthy control groups and HPT patients, respectively): (**a**) input; (**b**) BH-Net; (**c**) AlexNet; (**d**) ResNet34; (**e**) VGG16; (**f**) ResNet50.

To better demonstrate the classification performance of AlexNet, VGG16, ResNet34, ResNet50, and BH-Net, we used t-SNE (t-Distributed Stochastic Neighbor Embedding) to visualize the feature maps of each model before the fully connected layer [63]. t-SNE is a dimensionality reduction technique that maps high-dimensional data to a two-dimensional space. This approach enables us to observe how the features extracted by different models are distributed when processing BCG signals, and to compare the models’ ability to distinguish between categories in the learned feature space. The experimental results are shown in Figure 8. Figure 8a includes the input distribution, displaying the feature maps generated by the randomly initialized BH-Net. The converged feature maps of the BH-Net, AlexNet, ResNet34, VGG16, and ResNet50 models are shown in Figure 8b–f, respectively. We can see that, without training, the input data reveal randomness in the feature distribution. After training converges, the feature point distribution extracted by BH-Net shows a more distinct separation. This suggests that, compared to traditional deep learning models, the fusion of local and global characteristics in the proposed BH-Net improves the model discrimination ability, leading to a significant improvement in classification accuracy.

Also, the proposed network has the fastest inference time, fewest FLOPs, and smallest model size, suggesting that it is more efficient than the comparative models. HYP-Net is a recently proposed state-of-the-art network that is specifically designed for HPT detection with BCG signals. HYP-Net extracts features from time–frequency spectral images; therefore, although HYP-Net achieves better ROC-AUC values to some extent, it also requires much higher calculation complexity and inference time. On the other hand, the proposed BH-Net utilizes the features from the time domain sequences directly, demonstrating advantages in computational complexity and efficiency. Specifically, the inference time of BH-Net is 41.29 times faster than HYP-Net, and its FLOPs are only 1/182 of HYP-Net’s. Therefore, we can infer that the proposed BH-Net structure achieves a better trade-off between classification accuracy and calculation efficiency than the comparative network structures.

### 5.2. Analysis of Data Augmentation Results

**Figure 9:**
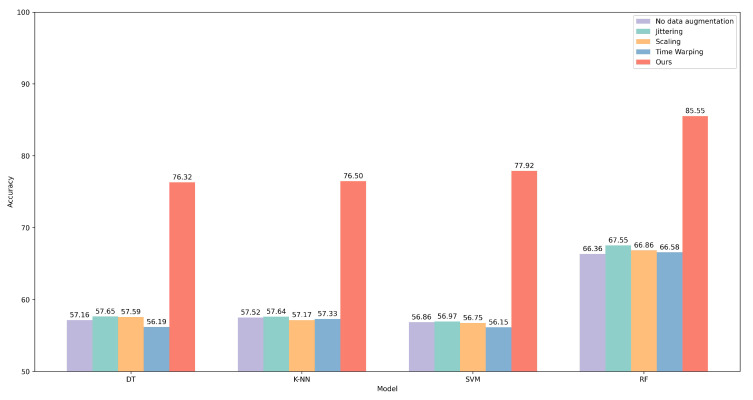
Accuracy results of different machine learning models under various data augmentation methods.

**Figure 10:**
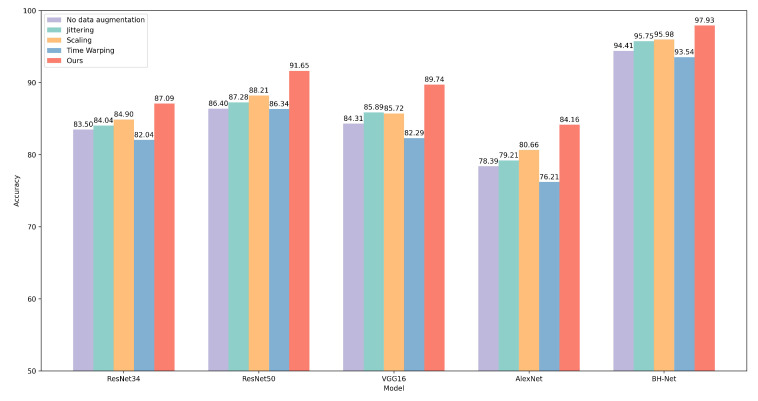
Accuracy results of different deep learning models under various data augmentation methods.

We selected three traditional time series data augmentation techniques, including Jittering, Scaling, and Time Warping [64]. We conducted experiments using these traditional augmentation methods, and the results are summarized in Figure 9 and Figure 10. For the three traditional data augmentation methods mentioned above, although they lead to some performance improvement in most models, the extent of the improvement is relatively small. In particular, the Time Warping method even causes performance degradation in certain models. This suggests that traditional data augmentation techniques may not be effective in improving feature representations of BCG signals. In contrast, the data augmentation method we proposed led to improved model accuracy, particularly for machine learning models, where the performance not only improved significantly but also enhanced the results of some deep learning models. This demonstrates the effectiveness of the proposed augmentation method.

Traditional augmentation techniques treat every part of the sample equally. Those regions that receive little attention from the model are still included in the data-set, which can undermine the calculation efficiency and accuracy. On the other hand, the proposed data augmentation approach of concatenating the J-peak neighborhoods is a selective scheme that can filter out the information that is most correlated to the HPT features, reflected by the heat-map. We define three metrics to quantitatively evaluate the correlation between the J-peak neighborhoods and the high-weight regions of the heat-map.

IOU: We define an arbitrary sampling point to be significant if the corresponding normalized heat-map weight exceeds a threshold value of 0.5. By accumulating the significant sampling points, we can define the significant region $S_{0.5}=\left\{x|\mathrm{heatmap}\left(x\right)>0.5\right\}$, and the IoU can be defined as

(9) $$IoU=\frac{S_{0.5}\cap N_{j-peak}}{S_{0.5}\cup N_{j-peak}}$$

where $N_{j}-peak$ represents the accumulated J-peak neighborhood regions of the sample. The metric IoU evaluates globally how the significant regions and the J-peak neighborhood regions are overlapped.

The Localization Rate (LR): We divide the BCG sample into segments according to the size of the J-peak neighborhood. For an arbitrary segment, if the number of significant sampling points exceeds 50% of the segment size, the segment is also considered to be significant. A hit label is assigned by a successful overlap of over 50% of the segment size of an arbitrary significant segment with any J-peak neighborhood; otherwise, a miss label is assigned. The Localization Rate is defined as

(10) $$LR=\frac{H}{H+M}$$

where *H* is the number of hit segments and *M* is the total number of miss segments. LR evaluates among all the significant regions how many of them are contained in the J-peak neighborhoods.

The Significant Rate (SR): For an arbitrary J-peak neighborhood region, if the number of significant sampling points exceeds 50% of the region size, a positive label is assigned; otherwise, a negative label is assigned. The Significant Rate is defined as

(11) $$SR=\frac{P}{P+N}$$

where *P* is the number of positive regions and *N* is the number of negative regions. The SR evaluates among all the J-peak neighborhood regions how many contain enough significant data points.

We report that, when we optimally chose the window size of the J-peak neighborhood to be 31 (a radius of 15 sampling points before and after the J-peak position), the IoU was 74.25%, the LR was 76.94%, and the SR was 82.15%. The choice regarding the neighborhood window size will be discussed in Section 5.3.

Therefore, we can conclude that there is a correlation between the network’s interested regions and the J-peak neighborhoods. When we select the J-peak neighborhoods from the BCG samples for data augmentation purposes, most of the selected regions contain high-weight data, and most of the high-weight regions are covered by the selected regions.

By selecting the high-weight regions in the data-set and discarding less-related regions, the feature representation can be enhanced; therefore, significant improvement in performance can be observed, as reported in Section 4.3.

This work demonstrates a method of employing a heat-map derived from the deep network training process. The heat-map can provide insight to build correlations between the high-weight regions and the features of the signal itself. Integrating such prior knowledge with other learning methodologies can improve their accuracy, thereby expanding their application range.

### 5.3. Parameter Selection

To determine the optimal radius for the J-peak neighborhood, we selected four different window sizes: 21, 31, 41, and 51. We computed the correlation metrics of IoU, LR, and SR for each of these sizes. The results are displayed in Table 6.

From Table 6, it can be observed that the Localization Rate and IoU metrics do not always show a consistent trend with the size of J-peak neighborhoods. When the window size increases from 21 to 31, both LR and IoU increase, but, when the window size further increases from 41 to 51, both metrics decrease. When the window size is too small, more high-weight regions will not be covered by the J-peak neighborhood. When the window size is too large, the J-peak neighborhood will contain more low-weight regions. Therefore, the LR and IoU will drop. In addition, as the window size increases, the SR gradually decreases, indicating that high-weight pixels are more densely clustered around the J-peak in the neighborhood. Therefore, the optimal balance point of the J-peak neighborhood window size should be selected to achieve the best data augmentation effect. We can see that the LR and IoU of window sizes 31 and 41 are similar and higher than the other options, suggesting better high-weight region coverage of the augmented data-set. The SR of window size 31 is larger than the window size of 41, which suggests that fewer low -eight regions are included in the augmented data-set. Therefore, in this study, we chose 31 as the optimal window size.

**Figure 11:**
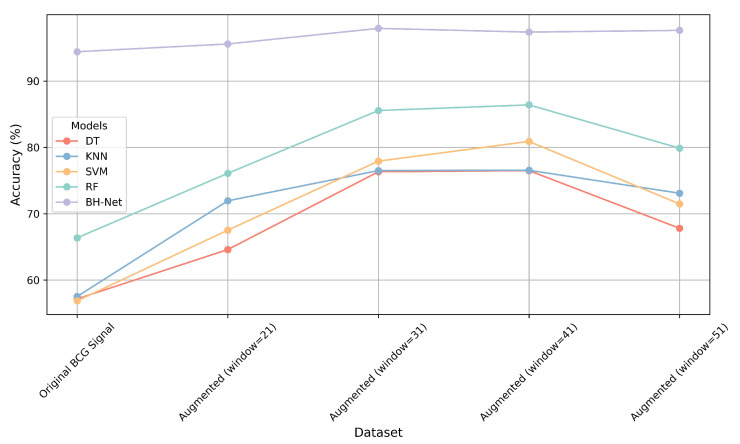
Classification results of different models.

We created four augmented BCG signal data-sets with the above-mentioned four distinct window sizes. We trained and tested traditional machine learning methods as well as the proposed BH-Net using both the original and augmented data-sets as the performance improvement in the machine learning models was the most significant when using the augmented data-set. The accuracy performance comparison is exhibited in Figure 11.

We can clearly see the influence exerted by the different window sizes to generate augmented data-sets on the classification results. A comparative analysis indicates that the data augmentation improves the classification accuracy compared to the original data-set, providing empirical support for the practicality of the data augmentation with the J-peak neighborhood data regarding HPT detection with BCG signals. We can see that, when the window size increases from 21 to 31, there is a significant improvement in the classification accuracy, particularly for the four machine learning methods. When the window size expands from 31 to 41, the accuracy performance is similar, and the improvement is relatively minor. When the window size increases to 51, the classification accuracy generally decreases.

We also summarized the efficiency metrics of the BH-Net model with different window sizes in Table 7. We can see that the inference time and FLOPs of BH-Net increase approximately linearly with the increase in the window size. When a larger window size is chosen, the length of the augmented sample will increase, thus increasing the calculation burden and computation time.

Therefore, we conclude that the window size of 31 is optimal to generate the augmented data-set. The performance comparison among the different window sizes agrees with the analysis of the correlation metrics, which justifies the selection of the J-peak neighborhood for data augmentation.

## 6. Conclusions

In this paper, we proposed an end-to-end lightweight neural network model named BH-Net for HPT detection via BCG signals. We rigorously evaluated the proposed network with a public data-set. We report that BH-Net demonstrated better performance regarding accuracy and efficiency compared to the state-of-the-art methods.

The heat-map of the proposed network indicates that the J-peak neighborhoods of the BCG signals received more attention from the proposed BH-Net in the HPT detection task. Based on this observation, we introduced the data augmentation scheme by selecting the J-peak neighborhood regions. With the augmented data-set, the accuracy of the BH-Net model was further improved, and significant performance improvement regarding traditional machine learning technologies and other deep learning networks can also be observed. 

## Figures and Tables

**Table 1:** Comparison of clinical characteristics.

	Hypertensive	Healthy Control Group
Number	61	67
Sex (Male/Female)	33/38	35/32
Age (years)	55.60±7.90	53.20±9.20
Heart Rate (bpm)	77.10±9.20	73.60±8.30
Body Mass Index (kg/m^2^)	24.30±3.60	23.70±3.30
Systolic Blood Pressure (mmHg)	155.60±11.20	112.10±15.70
Diastolic Blood Pressure (mmHg)	103.60±8.20	74.40±6.30

**Table 2:** Performance comparison of the proposed BH-Net with state-of-the-art methods.

Model	ROC-AUC	Accuracy	Macro-F1	Sensitivity	Specificity	Precision	Inference Time/s	Flops/G	Parameter Count/M
DT	57.49	57.16	56.98	57.00	56.20	56.98	-	-	-
KNN	61.48	57.52	57.37	58.34	56.88	58.62	-	-	-
SVM	62.71	56.86	52.39	53.95	54.55	53.90	-	-	-
RF	73.70	66.36	65.18	65.32	64.91	66.63	-	-	-
ResNet34	84.52	83.50	83.21	83.82	84.32	83.60	0.3589	1.1100	21.46
ResNet50	87.43	86.40	86.20	85.99	85.47	86.67	0.9091	1.2200	23.76
VGG16	85.58	84.31	84.20	84.19	84.45	84.23	0.5066	1.8400	134.27
AlexNet	79.38	78.39	75.06	75.75	76.08	74.39	0.1229	0.2283	56.99
Gupta et al. [58]	-	92.21	-	92.96	91.60	-	-	-	-
Rajput et al. [6]	-	86.14	87.00	87.60	84.31	-	-	-	-
HYP-Net [20]	99.24	97.65	97.50	96.77	98.48	98.36	2.0397	2.3100	0.62
Proposed	98.43	97.93	97.62	97.77	97.04	98.23	0.0494	0.0127	1.02

**Table 3:** Comparison of our work with state-of-the-art methods developed for automated detection of hypertension using BCG signals.

Study	Method	Input	Split Ratio (CV)	Accuracy
Rajput et al. [6]	Continuous wavelet transform, CNN.	Scalogram images	10-fold	86.14
Gupta et al. [58]	Multi-verse optimization, tunable Q-factor wavelet transform, and kNN.	Manually extracted features	10-fold	92.21
Gupta et al. [20]	CNN-based HYP-Net.	Spectral images	10-fold	97.65
Ours	CNN-based BH-Net with heat-map-guided augmentation.	Original signals	10-fold	97.93

**Table 4:** Classification results of machine learning methods with original BCG signal data-set and augmented BCG signal data-set.

Model	Original BCG Signal Data-Set					Augmented BCG Signal Data-Set				
	Accuracy	Macro-F1	Sensitivity	Specificity	Precision	Accuracy	Macro-F1	Sensitivity	Specificity	Precision
DT	57.16	56.98	57.00	56.20	56.98	76.32	76.29	76.03	76.41	76.30
K-NN	57.52	57.37	58.34	56.88	58.62	76.50	76.37	75.98	76.30	76.62
SVM	56.86	52.39	53.95	53.95	53.90	77.92	77.33	77.64	76.61	79.95
RF	66.36	65.18	65.32	65.32	66.63	85.55	85.91	85.46	85.89	85.56
BH-Net	94.41	94.35	95.65	91.40	94.28	97.93	97.26	97.77	97.04	98.23

**Table 5:** Classification results of deep learning models with original BCG signal data-set and augmented BCG signal data-set.

Model	Original BCG Signal Data-Set					Augmented BCG Signal Data-Set				
	Accuracy	Macro-F1	Sensitivity	Specificity	Precision	Accuracy	Macro-F1	Sensitivity	Specificity	Precision
ResNet34	83.50	83.21	83.82	84.32	83.60	87.09	86.96	86.86	86.12	87.11
ResNet50	86.40	86.20	85.99	85.47	86.67	91.65	91.60	91.69	91.21	91.54
VGG16	84.31	84.20	84.19	84.45	84.23	89.74	89.09	89.46	88.83	89.24
AlexNet	78.39	75.06	75.75	80.08	74.39	84.16	84.04	84.75	85.81	84.47
BH-Net	94.41	94.35	95.65	91.40	94.28	97.93	97.62	97.77	97.04	98.23

**Table 6:** Performance metrics with different window sizes and J-peak neighborhood radii.

Window Size	J-Peak Neighborhood Radius	Localization Rate (%)	IoU (%)	Significant Rate (%)
21	10	62.87	64.63	85.84
31	15	76.94	74.25	82.15
41	20	75.36	74.16	78.73
51	25	68.91	70.58	72.28

**Table 7:** Evaluation of BH-Net across different augmented data-sets.

Data-Set	Inference Time (s)	FLOPs (M)
Original data-set	0.0781	44.42
Augmented data-set (window = 51)	0.0547	21.86
Augmented data-set (window = 41)	0.0520	16.86
Augmented data-set (window = 31)	0.0494	12.71
Augmented data-set (window = 21)	0.0469	7.72

## Data Availability

Data are publicly available.
